# How to: Streamlining Pulsed Field Ablation-Based Pulmonary Vein Isolation Using 3D Mapping Without Fluoroscopy

**DOI:** 10.3390/jcm14124290

**Published:** 2025-06-16

**Authors:** Yannick Teumer, Lyuboslav Katov, Carlo Bothner, Wolfgang Rottbauer, Karolina Weinmann-Emhardt

**Affiliations:** Ulm University Heart Center, Ulm University, Albert-Einstein-Allee 23, 89081 Ulm, Germany; yannick.teumer@uniklinik-ulm.de (Y.T.); lyuboslav.katov@uniklinik-ulm.de (L.K.); carlo.bothner@uniklinik-ulm.de (C.B.); wolfgang.rottbauer@uniklinik-ulm.de (W.R.)

**Keywords:** pulsed field ablation, zero fluoroscopy, optimized workflow, 3D mapping, electrophysiology, atrial fibrillation, transesophageal echocardiography

## Abstract

**Background:** Pulsed field ablation (PFA) is a safe and effective method for pulmonary vein isolation (PVI) in atrial fibrillation (AF) patients. However, most first-generation PFA catheters are not integrated with 3D mapping systems, requiring fluoroscopy for guidance. The use of X-ray technologies, however, poses significant health risks to both patients and operating staff. Recently, a new variable-loop PFA catheter (VLC) with full 3D mapping integration allows for a novel fluoroscopy-free approach to PVI. In that regard, the aim was to evaluate and optimize a zero-fluoroscopy workflow for PVI using the VLC. **Methods:** Two workflows were described and compared: a conventional zero-fluoroscopy approach using a complete 3D left atrial map before ablation, and an optimized ‘mapping-on-the-fly’ approach that combines mapping and ablation into a continuous, real-time process for each pulmonary vein rather than performing them sequentially. **Results:** Forty-one pulmonary veins were successfully treated without fluoroscopy in 10 patients (20% female, median age 61 [IQR 55.5–66.8] years). Three patients underwent the conventional workflow, while seven received the optimized workflow. The ‘mapping-on-the-fly’ approach significantly reduced procedural time (median 68 vs. 144 min, *p* = 0.017) and left atrial dwell time (46 vs. 107 min, *p* = 0.016). No fluoroscopy-related complications occurred. **Conclusions:** PVI using the fully 3D-integrated VLC can be safely and efficiently performed without fluoroscopy. The optimized ‘mapping-on-the-fly’ workflow improves procedural efficiency.

## 1. Introduction

The advent of pulsed field ablation (PFA) represents a major advancement in atrial fibrillation (AF) treatment, offering selective myocardial targeting while preserving surrounding structures such as the esophagus, phrenic nerve, and pulmonary veins (PVs) [1]. This unique electroporation-based mechanism has led to significant improvements in procedural safety and efficiency in pulmonary vein isolation (PVI) for AF patients [2] while maintaining an efficacy profile comparable to traditional thermal ablation modalities such as radiofrequency (RF) and cryoballoon ablation [3].

**Figure 1:**
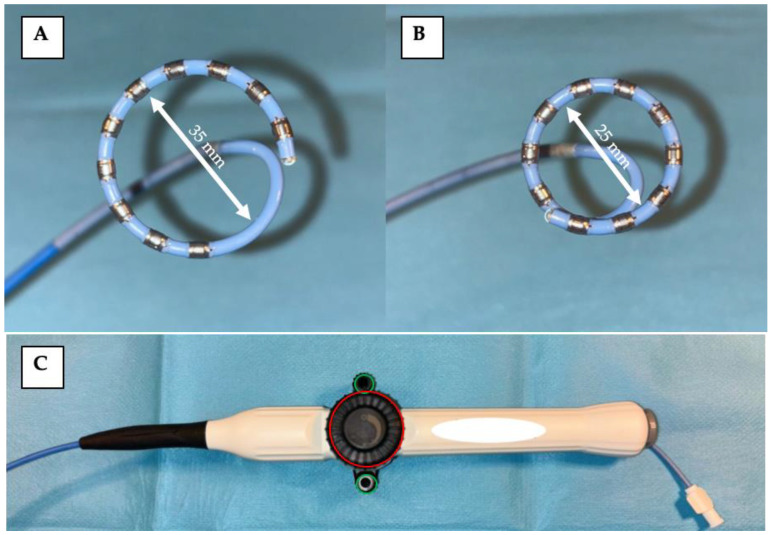
Illustration of the fully 3D mapping-integrated variable-loop pulsed field ablation catheter tip with 10 electrodes in two configurations: (**A**) open-loop (35 mm diameter) and (**B**) closed-loop (25 mm diameter). (**C**) The catheter handle, featuring a rotary knob (red circle) for adjusting the tip loop diameter and levers (green circle) for bidirectional flexing of the catheter tip.

As the clinical experience with PFA systems expands, workflow refinement and integration into modern electrophysiology labs have become key goals. While RF and some thermal single-shot ablation systems are already fully integrated with 3D electroanatomical mapping platforms, most commercially available PFA systems still rely on fluoroscopic imaging for catheter positioning and lesion delivery. This dependence on X-ray guidance not only increases cumulative radiation exposure for patients and staff but also limits the ability to leverage real-time anatomical feedback and spatial precision offered by modern mapping systems [3]. Recent efforts have focused on merging the advantages of PFA with the benefits of 3D mapping and fluoroscopy-free workflows, aiming to establish procedures that are simultaneously effective, safe, and radiation free. In this context, a novel variable-loop PFA catheter (Varipulse, 8.5F, Johnson & Johnson MedTech, New Brunswick, NJ, USA, VLC, Figure 1) has been developed. This system enables full 3D mapping integration and adjustable loop deployment, providing a versatile tool for personalized PVI strategies. The INSPIRE and ADMIRE trials have already validated the safety and efficacy of this catheter [4,5,6]. Importantly, the VLC’s compatibility with the Carto 3 mapping system now makes zero-fluoroscopy PVI procedures feasible, including full visualization of both the ablation catheter and the guiding sheath. Despite this innovation, published clinical data on zero-fluoroscopy PFA workflows remain limited, and optimized strategies for procedural streamlining are still under development.

In this study, we therefore present two distinct zero-fluoroscopy workflows using the VLC: a conventional approach involving complete left atrial mapping prior to ablation, and an optimized ‘mapping-on-the-fly’ technique in which each PV is mapped and ablated in a continuous sequence. Beyond describing these workflows in detail, we also report the first procedural data from our initial series of patients treated entirely without fluoroscopy, providing practical insight for centers aiming to implement similar workflows.

## 2. Materials and Methods

### 2.1. Study Design

This prospective, single-center study evaluated the feasibility of zero-fluoroscopy PFA using the novel 3D mapping integrated and irrigated VLC (Varipulse, 8.5F, Johnson & Johnson MedTech, CA, USA, Figure 1). Patients with symptomatic paroxysmal or persistent atrial fibrillation were enrolled. All procedures were performed in consecutively referred patients who met general eligibility criteria for PVI. No exclusion criteria were applied based on anatomical complexity. However, cases were scheduled on days with full team availability and device manufacturer technical support to ensure procedural quality during this early clinical experience. To minimize variability and accurately reflect the procedural learning curve, all procedures were performed by the same senior electrophysiologist experienced in TEE-guided and zero-fluoroscopy left atrial workflows. A consistent support team included an attending electrophysiologist and a TEE-proficient EP fellow, all of whom had undergone institutional training in fluoroless left atrial procedures. The first three patients were treated using a conventional mapping workflow. Due to longer-than-expected procedure and dwell times, an internal workflow audit prompted the implementation of an optimized ‘mapping-on-the-fly’ approach for the subsequent seven cases. The decision to switch was independent of patient anatomy or complexity and reflected a proactive effort to improve time efficiency and intra-procedural stability within the same patient population. All participants provided written informed consent for the ablation procedure. Data collection was approved by the institutional review board and conducted in accordance with the principles of the Declaration of Helsinki. The data were collected as part of the ATRIUM registry (German Clinical Trials Register ID: DRKS00013013).

### 2.2. Components of the Ablation System

The VLC (Varipulse, 8.5F, Johnson & Johnson MedTech, CA, USA) supports both high-resolution 3D electroanatomical mapping and PFA via 10 platinum–iridium electrodes integrated around an adjustable variable-diameter loop. The loop diameter can be continuously modified between 25 mm and 35 mm using a dedicated rotary control knob located on the catheter handle. The catheter also features dual-lever bidirectional steering, allowing precise manipulation and stable tissue contact within the left atrium. Each electrode can be selectively disabled to prevent overlap or interference during mapping or ablation.

For catheter tracking and 3D integration, the Carto 3 V8 electroanatomical mapping system (Johnson & Johnson MedTech, CA, USA) was used. Visualization of the sheath was enabled by the Vizigo steerable guiding sheath (8.5F, deflectable, Biosense Webster, Johnson & Johnson MedTech, CA, USA), which is fully 3D map integrated. This sheath is designed with embedded location sensors that allow full visualization and catheter guidance within the 3D mapping environment. It provides bidirectional deflection (180°), is compatible with PFA catheters, and was used to guide all left atrial manipulations after transseptal access.

PFA energy was delivered using the Truepulse PFA generator (Johnson & Johnson MedTech, CA, USA), a biphasic, high-voltage pulse generator specifically developed for nonthermal, irreversible electroporation-based cardiac ablation. The system delivers microsecond-scale biphasic waveforms in predefined energy trains optimized for myocardial selectivity while preserving adjacent noncardiac structures. The generator supports automated catheter recognition. Integrated safety features include impedance monitoring, pulse train interlocks, and fail-safe abort mechanisms.

### 2.3. Workflow

Following the induction of deep sedation for zero-fluoroscopy PVI utilizing the VLC, a transesophageal echocardiography (TEE) probe (CX50 system with an X7 TEE probe, Philips, Amsterdam, The Netherlands) is positioned at the mid-esophageal level to provide continuous imaging support. Subsequently, ultrasound-guided venipuncture of the right femoral vein is performed at two sites, in accordance with our established protocol [7].

After obtaining venous access, three-dimensional electroanatomical mapping of the right atrium is conducted using the VLC in combination with a 3D mapping system (Carto 3, V8, Johnson & Johnson MedTech, USA). VLC advancement into the right atrium and its manipulation is guided by intracardiac electrograms, TEE imaging, and the progressively generated right atrial map. Based on the reconstructed anatomy, a steerable, mapping-compatible 10-polar diagnostic catheter (Inquiry™ Steerable Diagnostic Catheter, Abbott, Lake County, IL, USA) is advanced into the coronary sinus under guidance from the mapping system.

The procedure proceeds with a TEE-guided transseptal puncture performed entirely without fluoroscopy and using a fixed transseptal sheath, as previously described by our group [7]. Upon successful access to the left atrium, intravenous heparin is administered with the aim of maintaining an activated clotting time exceeding 350 s, following the VLC manufacturer’s recommendations [4,8]. Subsequently, the fixed transseptal sheath is exchanged over wire for a 3D-mapping-visible sheath (Vizigo, 8,5F Johnson & Johnson MedTech, USA) under the guidance of TEE and the 3D mapping system. The 3D-mapping-visible sheath can be tracked from the right to the left atrium, thanks to the previously acquired right atrial map. Over this 3D-mapping-visible sheath the VLC is placed in the ostium of the left superior pulmonary vein (LSPV).

#### 2.3.1. Conventional Workflow

**Figure 2:**
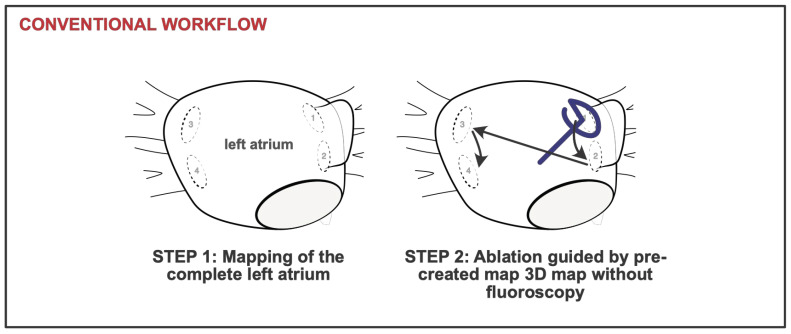
The diagram illustrates the conventional zero-fluoroscopy workflow using the variable loop catheter. In the conventional workflow, mapping and ablation are performed sequentially. 1, left superior pulmonary vein; 2, left inferior pulmonary vein; 3, right superior pulmonary vein; 4, right inferior pulmonary vein.

The conventional workflow begins with a detailed three-dimensional electroanatomical mapping of the entire left atrium, including all pulmonary veins (PVs), performed using the map-and-ablate VLC starting from the LSPV (Figure 2). This comprehensive map is then employed to guide PVI, which is carried out with the same catheter.

Ablation is initiated at the LSPV. At this location, two ostial and two antral energy applications are delivered, each consisting of a train of three pulses. Following each application, the catheter is rotated by 180 degrees, in accordance with the manufacturer’s recommendations [4,8], to ensure uniform and circumferential lesion formation. Ostial energy delivery was preferably performed using the closed-loop configuration, whereas antral energy deliveries employed the open-loop configuration (Figure 1). This protocol is systematically repeated for each of the remaining pulmonary veins.

**Figure 3:**
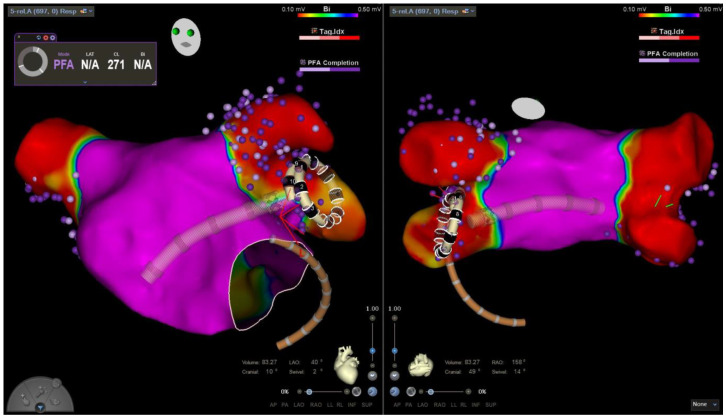
A screenshot of a left atrial map using the 3D mapping integrated map-and-ablate variable loop pulsed field ablation catheter (VLC). The screenshot shows a left atrial map in anterior view (**left**) and posterior view (**right**) after mapping and pulmonary vein isolation using the VLC. The VLC is positioned in the left inferior pulmonary vein, with purple dots indicating the locations of the VLC electrodes during pulsed field ablation energy delivery. White circles at the edges of the electrodes (black rings with white numbers) indicate optimal tissue contact.

After the ablation of each vein, entrance- and exit-block testing is conducted to confirm electrical isolation. In the event of persistent pulmonary vein signals detected on the PFA catheter, additional energy applications are administered at the corresponding sites until complete electrical isolation is achieved. After completing PVI, re-mapping of the left atrium was performed (Figure 2). During this re-map, PVI of all treated PVs was confirmed by entrance- and exit-block testing and electroanatomical mapping using the VLC (Figure 3).

#### 2.3.2. ‘Mapping-on-the-Fly’ Workflow

**Figure 4:**
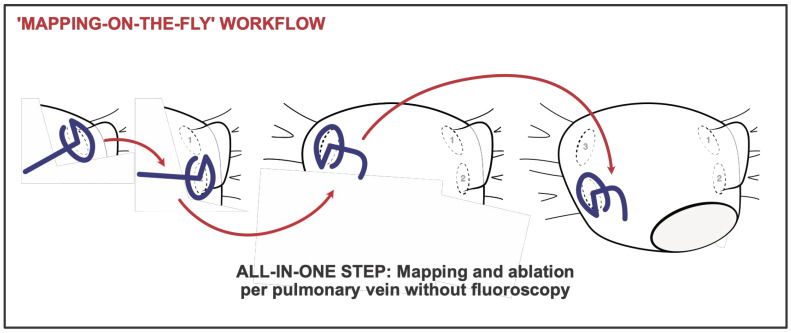
The diagram illustrates the continuous process of mapping and ablation in this zero-fluoroscopy ‘mapping-on-the-fly’ workflow using the variable loop catheter. In this workflow, the ostium of the respective pulmonary vein is mapped, and the pulmonary vein is ablated as soon as sufficient anatomy is present in the map. After completing the ablation of one pulmonary vein, mapping continues to the next pulmonary vein. 1, left superior pulmonary vein; 2, left inferior pulmonary vein; 3, right superior pulmonary vein; 4, right inferior pulmonary vein.

Unlike the conventional workflow, mapping and ablation were not performed separately but rather integrated into a continuous process (Figure 4). In the optimized ‘mapping-on-the-fly’ workflow, following transseptal access, the mapping catheter was positioned in the LSPV under TEE guidance. Subsequently, the LSPV was mapped during the withdrawal of the VLC from the PV. Following completion of the LSPV mapping and its transition to the left atrium, ablation was carried out in the same manner as in the conventional workflow. After ablation, entrance- and exit-block testing was performed at the LSPV. If PVI was achieved, mapping then proceeded from the LSPV ostium to the left inferior pulmonary vein (LIPV). After mapping the LIPV and its transition to the left atrium, ablation was performed in the same manner as at the LSPV. This process continued sequentially from the LIPV to the right superior pulmonary vein (RSPV) and then from the RSPV to the right inferior pulmonary vein (RIPV). Any additional PVs were incorporated into the mapping and ablation sequence. Upon completing PVI, re-mapping of the entire left atrium and PVs was conducted, starting from RIPV.

After completing PVI, re-mapping of the left atrium was performed (Figure 2). During this re-map PVI of all treated PVs was confirmed by entrance- and exit-block testing and electroanatomical mapping using the VLC.

### 2.4. Statistics

Statistical analysis was conducted using SPSS (version 29.0.1.0, IBM, Armonk, NY, USA). Descriptive and inferential statistics were applied based on the level of measurement. A *p*-value of <0.05 was considered statistically significant. Categorical variables were presented as frequencies and analyzed using either the chi-square test or Fisher’s exact test, as appropriate. Numerical variables were reported as medians with interquartile ranges (IQR) or as minimum and maximum values to illustrate distribution. Inferential testing was performed using either Student’s *t*-test or the Mann–Whitney U test, depending on data distribution.

## 3. Results

### 3.1. Patient Characteristics

A total of 10 patients (20% female; median age 61 (55.5–66.8) years) underwent PVI using the VLC in a zero-fluoroscopy setting. No significant differences in gender distribution were observed between groups. All patients had symptomatic paroxysmal or persistent atrial fibrillation. The median CHADS-VA score was 3 (1–3), indicating a moderate thromboembolic risk profile. Median body mass index (BMI) for the entire cohort was 26.2 (22.3–32.5) kg/m^2^.

Patients in the conventional mapping group and the mapping-on-the-fly group were similar in terms of age and AF type. However, the left atrial volume index was higher in the conventional group compared to the optimized workflow group (45.7 mL/m^2^ vs. 30.6 mL/m^2^; *p* = 0.033). Four patients (40%) had a reduced left ventricular ejection fraction (LVEF < 45%), all of whom were in the ‘mapping-on-the-fly group’. An overview of baseline characteristics is provided in Table 1.

### 3.2. Procedural Parameters

All procedures were completed successfully without the need of fluoroscopy. In total, 41 pulmonary veins were isolated across 10 procedures. Three patients underwent ablation using the conventional mapping-first workflow, whereas the remaining seven patients were treated with the optimized ‘mapping-on-the-fly’ approach.

The ‘mapping-on-the-fly’ strategy demonstrated a significant shorter median procedure duration of 68 (48–75) min compared to 144 (98–168) min in the conventional workflow group (*p* = 0.017). Likewise, the median left atrial dwell time was substantially reduced in the optimized group (46 (24–55) min) compared to the conventional group (107 (71–144) min; *p* = 0.016).

**Figure 5:**
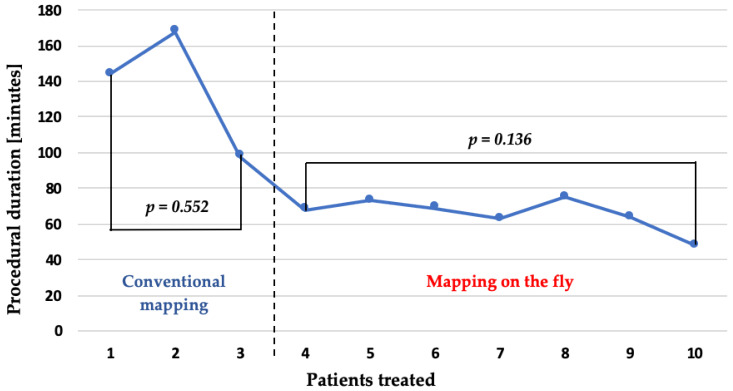
This figure illustrates the procedural duration for each treated patient. Patients 1–3 underwent the conventional workflow, while patients 4–10 were treated using the optimized ‘mapping-on-the-fly’ workflow.

Another notable procedural metric was the frequency of re-mapping: patients in the conventional group required a median of three re-maps, primarily due to map shifts caused by muscular contractions during PFA energy delivery. In contrast, patients in the ‘mapping-on-the-fly’ group required only a single final re-map to confirm isolation (3 (3-3) vs. 1 (1-1), *p* = 0.002). These temporal improvements are further visualized in Figure 5, which plots the individual procedure durations across the full patient series.

### 3.3. Complications

Importantly, there were no acute complications directly attributable to the zero-fluoroscopy technique. In particular, no cases of cardiac perforation, pericardial effusion, tamponade, vascular access injury, or emergency conversion to fluoroscopy were observed. All transseptal punctures, sheath exchanges, and catheter manipulations were safely completed using transesophageal echocardiography and 3D mapping guidance alone.

One patient experienced a postprocedural ischemic event approximately 10 h after ablation. The patient developed unilateral paresthesia of the left cheek and transient fine motor impairment of the left hand. MRI revealed embolic ischemic lesions localized to the right mesencephalon, consistent with the patient’s symptoms. The event was managed by immediate transfer to our stroke unit with a mild outcome. It occurred despite adequate periprocedural anticoagulation: the patient had been on uninterrupted oral anticoagulation for over 21 days, and intra-procedural ACT was maintained above 350 s with full-dose heparin. No additional thromboembolic, bleeding, or procedural adverse events were recorded.

## 4. Discussion

Following the ALARA (As Low As Reasonably Achievable) principle, the goal of zero fluoroscopy was successfully achieved in all 10 patients using the presented workflows [9]. Although the absolute risk of radiation-induced malignancies or other adverse effects from a single ablation procedure may be low, cumulative lifetime exposure for patients undergoing repeated procedures, as well as for operators and staff, remains a major concern [9,10]. Another key reason for eliminating radiation exposure in electrophysiological procedures is to mitigate stochastic effects [10,11]. Given the limited available evidence on completely zero fluoroscopy AF ablation procedures [11], our workflow represents a meaningful reduction in radiation exposure compared to other PFA-PVI workflows, where fluoroscopy durations range between 4.4 ± 3.1 and 21.1 ± 11.0 min [3,4,6,12].

Overall, the procedural duration in this study was within the documented range for PVI using the VLC [4,6,13,14], despite the implementation of a zero-fluoroscopy workflow and additional safety measures during transseptal puncture.

In our study, TEE was selected to guide the zero-fluoroscopy workflow due to its institutional availability and the procedural team’s longstanding experience in using TEE for left atrial access and navigation. It must be noted that the use of TEE in such workflows requires a dedicated operator proficient in both imaging and electrophysiological anatomy, particularly in the context of transseptal puncture and intracardiac catheter manipulation without fluoroscopic support. In centers where available, intracardiac echocardiography (ICE) offers a valuable alternative. ICE allows for real-time imaging of the interatrial septum, catheters, and lesion formation, and can be integrated into zero-fluoroscopy workflows to support both safety and efficacy. Future developments in ICE-guided navigation may further enhance the reproducibility of fluoroless PFA workflows [15].

Notably, procedural times decreased meanfully over the first 10 cases, demonstrating a steep learning curve for zero-fluoroscopy PVI with the VLC. This learning curve was comparable to that observed with other PFA systems [16,17,18]. From our point of view, beyond operator experience, an additional factor contributing to shorter procedure times was the shift from a conventional to a ‘mapping-on-the-fly’ workflow. Muscular contractions during PFA delivery are intrinsic to the electroporation mechanism and can cause transient anatomical distortions that affect electroanatomical map integrity. In our experience, this manifested as ‘map shifts’ during the conventional workflow, necessitating repeated re-mapping to maintain anatomical accuracy. The optimized ‘mapping-on-the-fly workflow’ mitigated this issue by mapping and immediately ablating each PV before moving to the next PV, minimizing the temporal gap between mapping and ablation. Consequently, the median number of required re-maps per procedure was reduced from three to one, with the final re-map used solely to assess ablation success. While no quantitative measurements of contraction severity were conducted in this study, future research should aim to systematically characterize muscular responses to PFA and identify predictive factors for significant map shift, potentially enabling individualized preprocedural risk stratification.

Our workflow modification after three cases reflects a common clinical process of procedural adaptation in the early phase of novel technologies. Rather than pre-selecting patients, the same operator and team iteratively refined the approach in pursuit of both safety and procedural efficiency.

The combination of PFA with 3D mapping technology enables not only zero-fluoroscopy procedures but also provide a more accurate assessment of lesion quality, potentially improving procedural outcomes [19,20]. Integrated 3D mapping allows for more precise localization of PFA lesions and facilitates early identification of potential isolation gaps in the pulmonary veins, which is more challenging with fluoroscopy-guided PFA-PVI systems.

An additional advantage of PVI with the VLC is the ability to assess tissue contact using the Tissue Proximity Indication feature, in addition to the operator’s tactile feedback. Tissue contact is crucial for optimal and transmural lesion formation during PFA [21,22,23]. Unlike fluoroscopy-guided PFA systems, where lesion formation is often inferred indirectly, 3D mapping integration provides a more accurate assessment of lesion quality, potentially improving procedural outcomes [19,20].

Furthermore, the integration of 3D mapping into PFA workflows also aligns with the evolving paradigm of atrial cardiomyopathy-driven ablation strategies. Within this framework, electroanatomical mapping can support individualized lesion set design based on patient-specific atrial remodeling patterns. Future developments may combine mapping data with fibrosis quantification from delayed-enhancement MRI and circulating biomarkers to stratify patients and guide therapy more precisely [24]. Such integration may ultimately enhance long-term efficacy and patient selection for substrate-based ablation strategies.

In this first-in-experience cohort, we observed no zero-fluoroscopy-associated complications such as cardiac perforation, pericardial effusion, tamponade, inadvertent transseptal misplacement, or vascular complications. The use of TEE, in conjunction with real-time 3D mapping and a trained procedural team, enabled precise catheter navigation and sheath control throughout the procedure. Importantly, no conversions to fluoroscopy or rescue imaging were required. By reduction in catheter movements within the left atrium following the ‘mapping-on-the-fly’ approach, this workflow optimization may not only improve procedural efficiency but might also enhance periprocedural safety.

One patient experienced a postprocedural ischemic stroke, representing a serious but isolated event in this early experience. The stroke occurred approximately 10 h post-ablation and manifested with subtle left-sided sensory and motor deficits. Imaging confirmed embolic infarcts in the right mesencephalon with a mild outcome after immediate transfer to our stroke unit. The patient had been on uninterrupted oral anticoagulation (OAC) for more than three weeks prior to the intervention, and intra-procedural ACT was rigorously maintained >350 s using intravenous heparin according to the manufacturer’s recommendations. TEE was performed prior to transseptal puncture to exclude intra-atrial thrombus. Both the catheter and the sheaths used in the procedure were sent to the manufacturer for formal evaluation. The investigation did not reveal any device-related issues or technical malfunctions. Consequently, the stroke is considered an unforeseeable adverse event rather than a device-associated complication.

### Limitations

This single-center feasibility study is limited by its small cohort size and non-randomized design. As such, conclusions regarding procedural efficacy and safety must be interpreted with caution. The 3:7 split between the conventional and optimized workflow reflects a deliberate adaptation after internal analysis of procedural durations. Furthermore, a temporary halt in catheter usage during the study period, following a global safety communication and review process by the manufacturer, limited the number of cases that could be included. Durability of PVI, a central endpoint in any ablation strategy, could not be evaluated within the scope of this feasibility study. Although achieving zero fluoroscopy aligns with the goal of the ALARA principle, zero-fluoroscopy workflows are limited in patients with pacemakers and implantable cardioverter-defibrillator leads. These leads must be carefully considered during atrial procedures to prevent potential damage or displacement caused by sheath or catheter movement. As a result, fluoroscopy is still required in these cases.

## 5. Conclusions

In this single-center feasibility study, PFA-PVI was performed safely and efficiently without the need for fluoroscopy using the novel 3D-mapping integrated VLC. Procedural efficacy of zero-fluoroscopy PFA-PVI can be enhanced by adopting a ‘mapping-on-the-fly’ workflow.

## Figures and Tables

**Table 1:** Baseline characteristics of the treated cohort.

Parameter	Total n = 10	Conventional Mapping n = 3	Mapping on the Fly n = 7	*p* Value
Age, median (IQR) [years]	61 (55.5–66.8)	63 (46.0–63.0)	60 (56.0–62.0)	0.732
Female patients, n (%) Male patients, n (%)	2 (20.0) 8 (80.0)	1 (33.3) 2 (66.7)	1 (14.3) 6 (85.7)	0.490
Body mass index, median (IQR) [kg/m^2^]	26.2 (22.3–32.5)	24.7 (21.9–24.7)	27.9 (22.4–35.5)	0.667
Left-atrial volume index, median (IQR) [ml/m^2^]	38.3 (28.9–43.0)	45.7 (38.5–45.7)	30.6 (27.2–38.3)	0.033
Left-atrial diameter [mm], median (IQR)	46 (42–50)	45 (42–45)	49 (42–51)	0.252
CHADS-VA-Score, median (IQR)	3 (1–3)	1 (1–1)	3 (1–4)	0.724
Paroxysmal atrial fibrillation, n (%) Persistent atrial fibrillation, n (%)	5 (50) 5 (50)	2 (66.7) 1 (33.3)	3 (42.9) 4 (57.1)	0.490
Reduced LVEF ^1^, n (%)	4 (40.0)	0 (0.0)	4 (57.1)	0.232

^1^: left-ventricular ejection fraction defined as <45%.

## Data Availability

The data presented in this study are available on request from the corresponding author. The data are not publicly available due to data privacy law.

## Abbreviations

The following abbreviations are used in this manuscript:

ALARA	As low as reasonably achievable
ICE	Intracardiac echocardiography
LIPV	Left inferior pulmonary vein
LSPV	Left superior pulmonary vein
OAC	Oral anticoagulation
PFA	Pulsed field ablation
PV	Pulmonary vein
PVI	Pulmonary vein isolation
RIPV	Right inferior pulmonary vein
RSPV	Right superior pulmonary vein
TEE	Transesophageal echocardiography
VLC	Variable loop catheter
